# When Cholangitis Reveals Liver Involvement in Hereditary Hemorrhagic Telangiectasia: A Case Report

**DOI:** 10.7759/cureus.85069

**Published:** 2025-05-30

**Authors:** Soumya El Graini, Siham Oukassem, Hamza Retal, Ittimade Nassar, Kaoutar Imrani

**Affiliations:** 1 Radiology, Ibn Sina University Hospital, Mohammed V University, Rabat, MAR

**Keywords:** angiocholitis, arterial liver disorder, biliary dilatation, biliary ischemia, hereditary hemorrhagic telangiectasia

## Abstract

Hereditary hemorrhagic telangiectasia (HHT) syndrome is a rare autosomal dominant hereditary disorder characterized by multiple arteriovenous malformations (AVMs), resulting from capillary dysplasia, responsible for arteriovenous shunting. It can affect various organs, including the lungs, liver, and central nervous system. Clinical manifestations are epistaxis, telangiectasias, and complications of visceral AVMs. Biliary involvement is uncommon and typically results from biliary ischemia. This can lead to complications such as cholangitis, biliary strictures, cysts, necrosis, and bilomas. Although less frequent, biliary complications may present similarly to bile duct obstruction or sepsis and necessitate careful management. We report the case of a 55-year-old patient with a history of unexplored epistaxis who presented with hepatic colic. Clinical and laboratory findings revealed telangiectasias, cholestasis, and an inflammatory syndrome. Imaging revealed hepatic artery dilatation, multiple AVMs in hepatic telangiectasias and a porto-hepatic shunt, and segmental cystic dilation of the bile ducts. The patient was treated for cholangitis and remains under surveillance. This case highlights the importance of multimodal imaging in evaluating hepatic involvement in patients with HHT. Indeed, a combination of ultrasound, multiphasic CT, and dynamic MRI helps reveal not only typical vascular abnormalities, including hepatic artery dilatation, telangiectasias, and arteriovenous shunts, but also biliary involvement. Regular follow-up is necessary to tailor management and prevent potential complications associated with this hereditary vascular syndrome.

## Introduction

Hereditary hemorrhagic telangiectasia (HHT) syndrome is a rare autosomal dominant hereditary disorder characterized by multiple arteriovenous malformations (AVMs), resulting from capillary dysplasia, responsible for arteriovenous shunting [1]. These lesions can be small and punctate, corresponding to telangiectasias, and are located on the skin surface or mucous membranes, particularly in the oral, nasal, labial, or lingual areas, or on the face. Larger AVMs most often occur in the lungs, liver, digestive system, or brain [1]. In addition to these two manifestations, the syndrome is characterized by the occurrence of epistaxis, leading to long-term anemia [2]. The diagnosis is based on the Curaçao criteria and confirmed by genetic testing, which can help clarify the genetic status of asymptomatic at-risk family members [2,3].

Hepatic AVMs are common in HHT, though frequently asymptomatic. When symptomatic, they may lead to high-output cardiac failure, portal hypertension, or biliary complications. Biliary manifestations, while rare, can reflect ischemic injury due to altered hepatic blood flow and vascular shunting. There is no curative treatment for HHT; management focuses on symptom control and organ-specific complications [2].

This is a case of a 55-year-old patient who presented with abdominal pain, and in whom clinical, biological, and radiological examinations revealed angiocholitis due to biliary ischemia secondary to HHT syndrome.

## Case presentation

A 55-year-old male patient, with a history of recurrent episodes of epistaxis that had not previously been investigated, was admitted for right upper quadrant pain, which evolved over the past two months.

**Figure 1:**
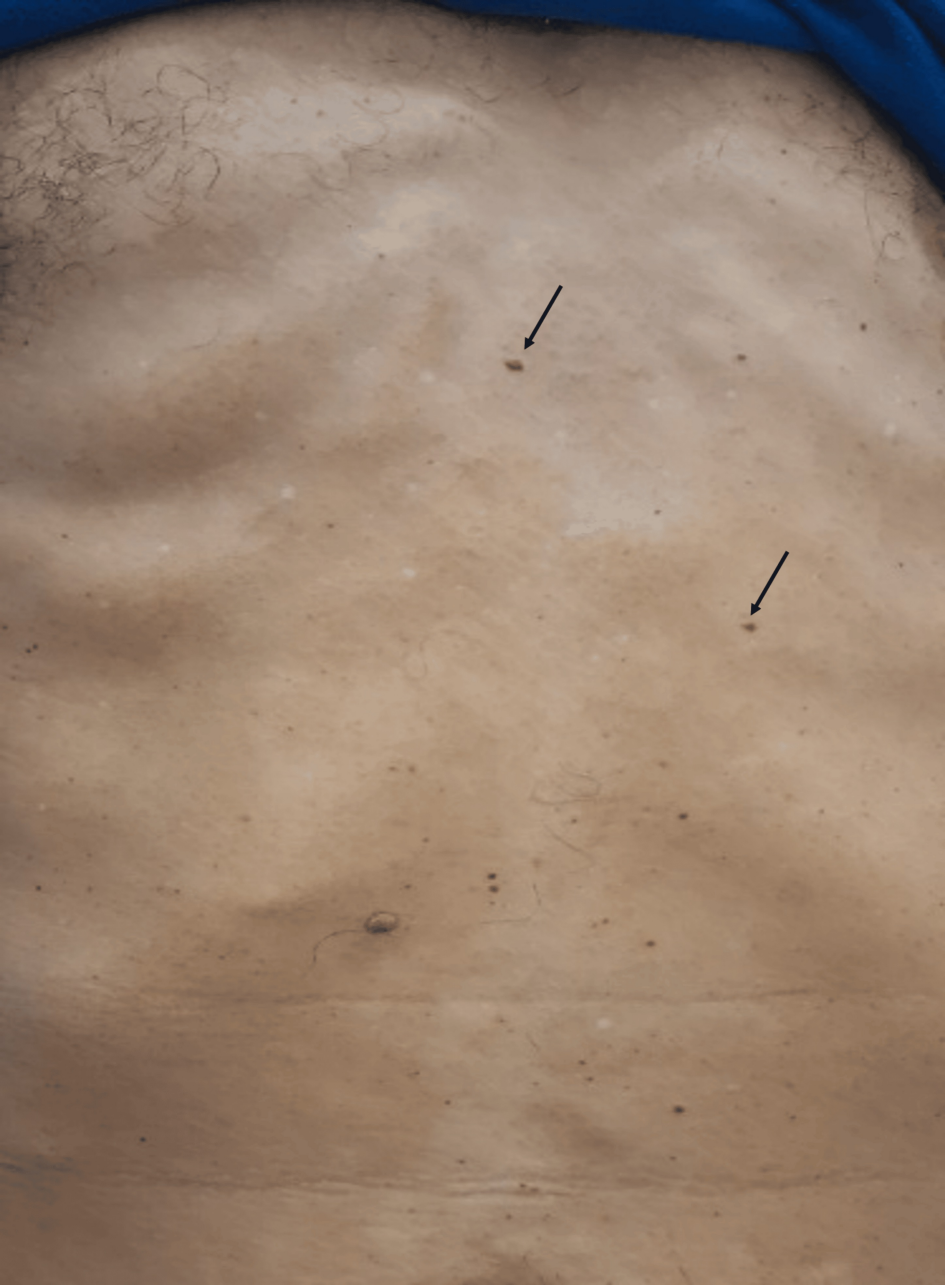
Clinical examination showing telangiectasia (arrows)

Clinical examination revealed a conscious and well-oriented patient with hemodynamic and respiratory stability. However, he was febrile (38.5°C) and abdominal examination revealed mild tenderness in the right upper quadrant, along with the presence of a few cutaneous telangiectasias (Figure 1).

**Figure 2:**
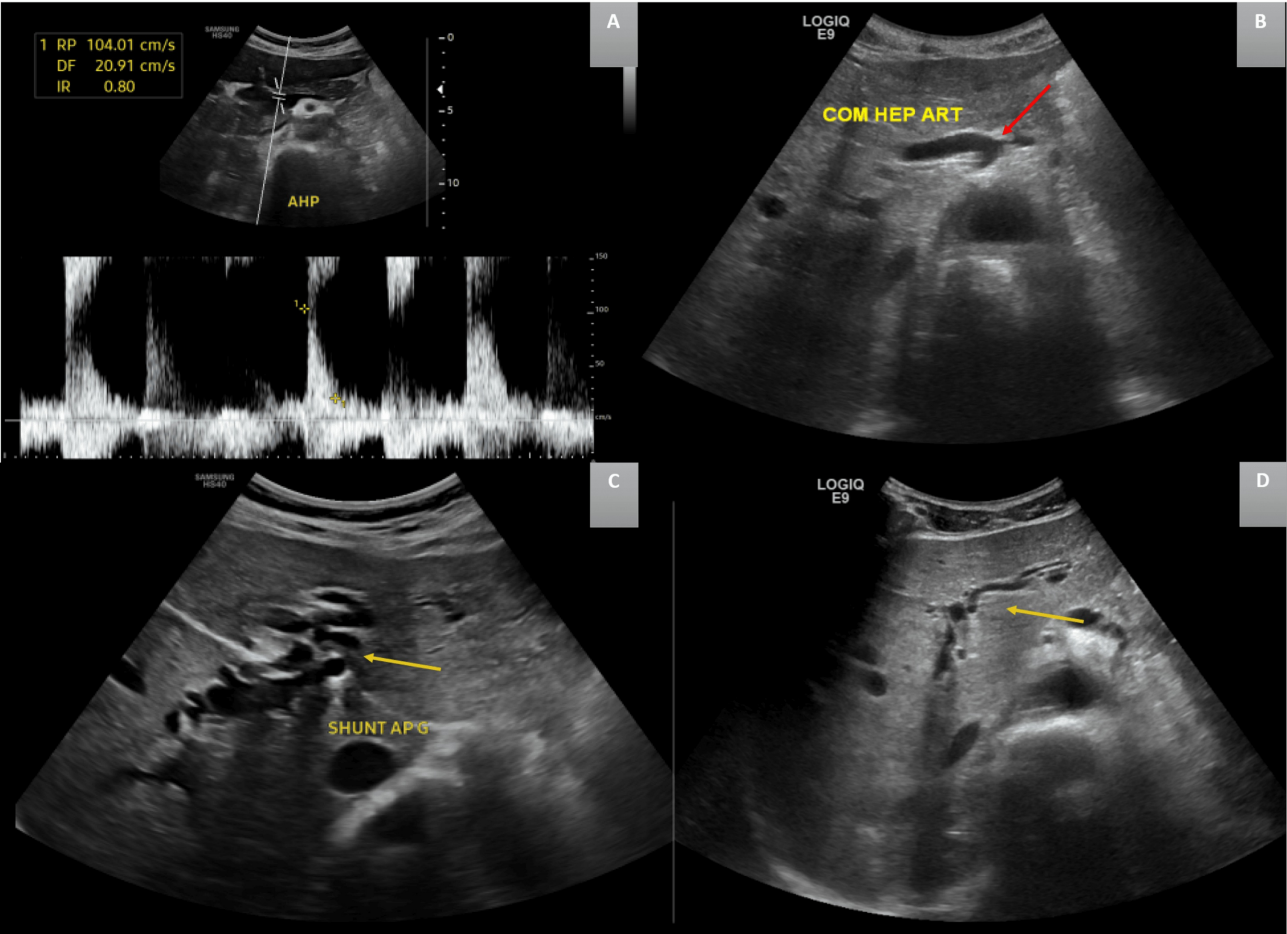
: Ultrasound images (A) Color Doppler revealing high velocity (104 cm/second) and a high resistance index in the hepatic artery measuring 0.8; (B) Dilation of the hepatic artery (red arrow); (C, D) Tortuous intrahepatic tubular structures and arterio-portal shunts (yellow arrows)

An abdominal Doppler ultrasound was conducted, revealing an enlarged hepatic artery, with increased flow velocities measuring 104 cm/second and a high resistance index measuring 0.8. Multiple tortuous structures were observed, suggestive of arteriovenous communications between the hepatic arteries and the portal veins. The portal venous system showed no evidence of thrombosis or obstruction. There was no dilatation of the extrahepatic bile ducts. The liver parenchyma appeared heterogeneous, and oblong, cystic, anechoic lesions with fluid-fluid levels were noted in segments VII and VIII of the liver (Figure 2).

Laboratory tests revealed signs of inflammation, including hyperleukocytosis (13,000/mm³) and elevated C-reactive protein (CRP) at 90 mg/L, as well as cholestasis characterized by markedly elevated alkaline phosphatase (350 U/L) and gamma-glutamyl transferase (220 U/L). Total bilirubin was elevated at 0.060 mmol/L, with a predominance of direct (conjugated) bilirubin at 0.048 mmol/L. Aminotransferases were mildly increased, with aspartate aminotransferase (AST) at 65 U/L and alanine transaminase (ALT) at 70 U/L.

**Figure 3:**
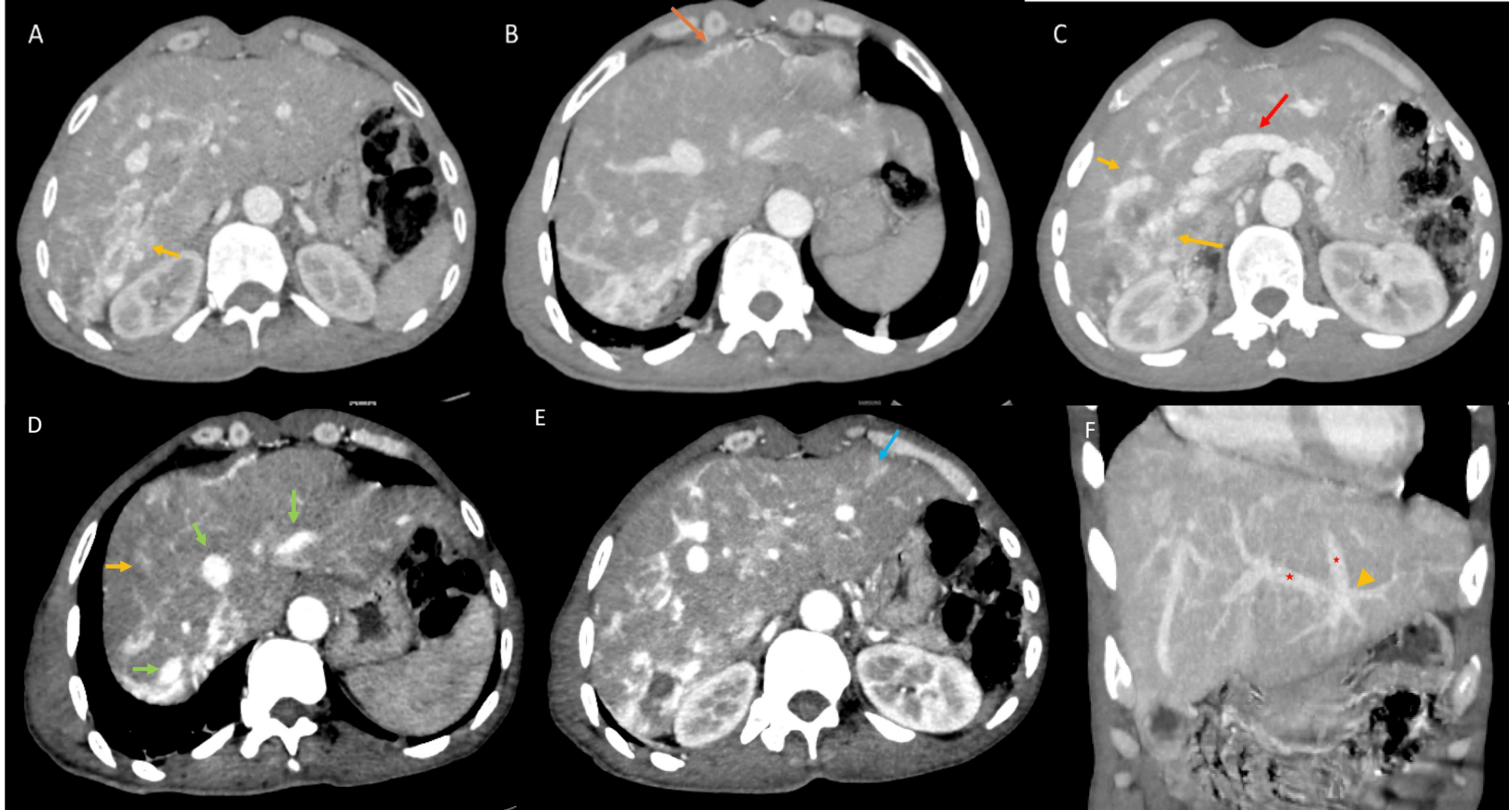
Triphasic hepatic CT-scan in axial (A,B,C,D,E) and coronal views (F) showing signs of liver involvement in hereditary hemorrhagic telangiectasia (A) Arterial phase scan showing a supra-centimetric, round, hypervascular lesion suggestive of telangiectasia (yellow arrow); (B, E) Arterial phase scan showing an early portal vein enhancement, suggesting an arterio-portal shunt, which appears as wedge-shaped areas (orange and blue arrows, respectively); (C) Arterial phase scan showing enlargement of the hepatic artery (red arrow); (D) Arterial phase scan showing early enhancement of the hepatic vein (green arrows) in the arterial phase, indicating the presence of arterio-venous shunts; (F) Portal phase scan showing communications between portal venous branches and hepatic veins (yellow arrowhead) suggest porto-venous shunts.

**Figure 4:**
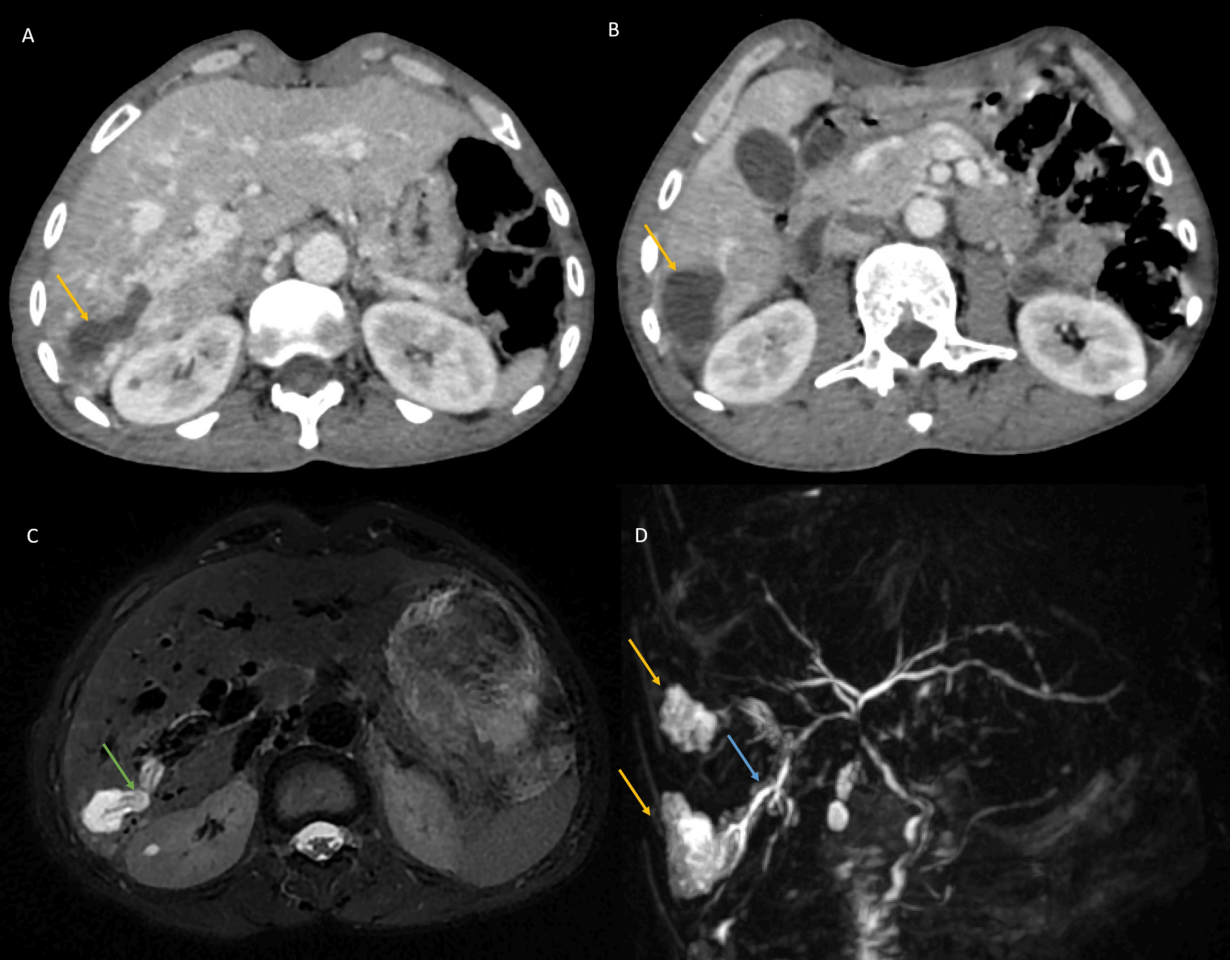
Axial portal phase CT-scan (A, B) and liver MRI in axial T2W FS (C) and coronal T2 RARE (D) showing biliary involvement in hereditary hemorrhagic telangiectasia There are cystic lesions in the VI and VII segments (yellow arrows), communicating with biliary trees (blue arrow), with fluid-fluid level (green arrow) and no enhancement after injection, suggestive of a bilomas. T2W FS: T2 weighted fat saturated; RARE: rapid acquisition with relaxation enhancement

Further evaluation with CT scan and liver MRI revealed significant dilatation of the hepatic artery, multiple arteriovenous shunts, and early abnormal enhancement of the hepatic veins during the arterial phase. The CT scan also identified multiple hypervascularized nodules scattered throughout the liver parenchyma, consistent with telangiectasias, which were confirmed by dynamic contrast-enhanced MRI (Figure 3). The cystic lesions in segments VII and VIII appeared to communicate with the intrahepatic bile ducts, with no signs of upstream obstruction or abnormalities of the extrahepatic bile ducts (Figure 4). There were no signs of hepatic neoplasia or chronic liver disease.

The diagnosis of HHT was established based on the Curaçao criteria, which include recurrent spontaneous epistaxis, multiple cutaneous telangiectasias, and visceral vascular malformations. In this case, the patient presented with recurrent epistaxis, visible cutaneous telangiectasias, and hepatic vascular malformations, fulfilling three of the four diagnostic criteria and confirming a definite diagnosis of HHT. This likely led to ischemia of the bile ducts, complicated by bilomas that became infected, resulting in cholangitis. Thoracic imaging showed cardiomegaly without evidence of pulmonary vascular malformations. Brain MRI did not reveal any abnormalities.

The patient received antibiotic therapy for cholangitis, which led to symptom improvement. He is currently under medical surveillance, pending potential liver transplantation.

## Discussion

HHT syndrome is a rare hereditary autosomal dominant disorder, characterized by systemic vascular dysplasia [2,4]. Its prevalence is estimated between 1/5000 and 1/8000, with higher frequency in some geographical areas, such as the United States and western and northern Europe [2,4].

In 90% of cases, it is caused by a heterozygous mutation in two genes, endoglin (*ENG*) and activin-like receptor kinase (*ALK1*), responsible for regulating angiogenesis, ensuring endothelial migration and proliferation [2]. This impairment also leads to a disruption of leukocyte infiltration, decreasing the inflammatory response and, consequently, vascular remodeling, making the person susceptible to infections. Furthermore, there are coagulation disorders, particularly a high risk of bleeding, which becomes prolonged and profuse [2]. In other cases, pathogenic variants related to HHT have been described involving other genes, such as *MADH4*, *GDF2*, and *RASA-1* [2].

Clinically, this vascular dysplasia manifests as epistaxis, visceral AVM, and telangiectasias [4]. Indeed, epistaxis is recurrent and spontaneous in 50% of patients at the age of 20, and in more than 90% at 40, responsible for anemia [2]. Telangiectasias are also commonly found and correspond to malformations of small arterioles and venules at the level of capillary beds. They appear as pinpoint lesions and can be located on the lips, face, tongue, fingers, as well as in the gastrointestinal tract and nasal or oral mucosa [2]. AVMs can be located in the lungs, brain, liver, or gastrointestinal tract. They may be asymptomatic or present with symptoms depending on the extent of the involvement [2].

Hepatic involvement has been reported to occur in approximately 41-71% of cases [2,4], but only about 8% of affected patients present with symptoms [5]. It’s characterized by shunts that can occur at different levels: hepato-portal (between the hepatic artery and the portal vein), arterio-venous (between the hepatic artery and the hepatic vein), and porto-venous (between the portal vein and the hepatic vein) [2]. These shunts can lead to three clinical presentations described by Garcia-Tsao et al., depending on the dominant type of shunt: high-output cardiac failure (the most common), portal hypertension, and biliary disease [5].

Cardiac failure is primarily due to arteriovenous shunts, leading to excessive venous return to the heart and right heart failure [4]. For portal hypertension, it can be secondary to increased blood flow due to an arterio-portal shunt, but is more often secondary to increased intrahepatic resistance caused by regenerative nodular hyperplasia [4]. It causes splenomegaly, ascites, and gastroesophageal varices.

Finally, since the biliary tract is solely supplied by the hepatic artery, arteriovenous shunts can cause a diversion of blood flow in the peribiliary plexus, leading to biliary ischemia and necrosis [3,4]. This is often associated with cholestasis, stenosis, biliary extravasation forming bilomas, and occasionally cholangitis [3]. Patients tend to present with symptoms of cholangitis or similar to bile duct obstruction or sepsis [4,6]. Intra-biliary hematomas causing inflammation of the biliary tract are rare [6]. This was the case for our patient, who presented with bilomas revealing biliary ischemia due to hepatic arteriovenous shunting.

In some cases, hepatic encephalopathy and mesenteric ischemia may also be observed in HHT. The first is a result of porto-hepatic shunting, while the second is due to diversion of mesenteric flow through the hepatic artery [2,4]. Focal or diffuse hepatic vascular malformations (HVMs) also lead to increased arterial perfusion and reduced portal perfusion, along with heightened regenerative activity, which can result in the formation of focal nodular hyperplasia [3].

Likely, all three types of shunts occur simultaneously, with one dominant type causing the main clinical subtype. However, the dominant type of shunt may change over time, which could explain overlapping clinical presentations and the transition from one clinical type to another [4].

AVM can also occur in the lungs in 50% of cases, manifesting as hemoptysis or hemothorax [2]. Gastrointestinal involvement, estimated at 30%, primarily leads to bleeding, and AVMs are most often found in the stomach and small intestine (especially the duodenum) and rarely in the colon [2]. In the nervous system, various types of AVMs exist, and the risk of bleeding varies [2].

The diagnosis is based on the Curaçao criteria, which include four parameters: (i) spontaneous and recurrent epistaxis, (ii) cutaneous or mucosal telangiectasia, (iii) visceral arteriovenous lesions in an internal organ, and (iv) family history [2]. It is considered probable or suspected if at least two of these criteria are present and certain if three are present [2,3]. Our patient presented with recurrent epistaxis, cutaneous telangiectasias, and hepatic vascular malformations, fulfilling three of the Curaçao criteria, thus confirming the diagnosis of HHT.

Imaging plays a fundamental role in the management of these patients, relying on ultrasound combined with Doppler, as well as abdominal CT angiography or hepatic MRI angiography, to determine the predominant type of vascular malformation [2,3]. Color Doppler ultrasound is a non-invasive, cost-effective screening tool for HVMs, with a sensitivity of 86-97% and a specificity of 100% [3]. It evaluates key parameters such as the hepatic artery and the presence of vascular shunts. HVMs have a prevalence of 32-72% [3], and ultrasound plays a central role in their diagnosis, staging, and follow-up. Characteristic findings of HHT include hepatic artery dilation (>4 mm), reversed hepatic-to-splenic artery diameter ratio, and peak hepatic artery velocity >100 cm/second [6]. Additional features such as peripheral subcapsular Doppler "spots" with high velocity and low resistive index may indicate small peripheral malformations [6]. Other suggestive signs include tortuous intrahepatic tubular structures and arterio-portal or arterio-systemic shunts, which can cause arterialization, dilation, and turbulent portal or hepatic vein flow. In advanced HHT, progressive vascular abnormalities may lead to structural liver changes and fibrosis, resulting in pseudocirrhosis [7].

On CT or MRI, diagnostic clues include significant enlargement of the hepatic artery, more pronounced than other pathologies such as hepatocellular carcinoma, hemangioma, or cirrhosis [3,4]. Telangiectasias are the most common hepatic lesions, appearing as hypervascular, often supra-centimetric round lesions that enhance in the arterial phase and resemble liver parenchyma in the late phase [3,4]. They may merge to form a large vascular mass. Arteriovenous shunts, particularly between the hepatic artery and veins, are best visualized during the arterial phase with early hepatic vein enhancement [3]. Early portal vein enhancement suggests an arterio-portal shunt, appearing as wedge-shaped areas. Porto-venous shunts are rarer and harder to diagnose but may be seen in the portal phase with direct communication between portal venous branches and hepatic veins. Benign regenerative nodules, resembling HNF-like lesions, can be differentiated from telangiectasias using hepatospecific contrast agents in MRI [3].

Our case reinforces the importance of systematic imaging in HHT patients, particularly those with hepatic symptoms. It highlights the coexistence of multiple hepatic manifestations, including biliary involvement, which is considered rare. It should be noted that liver biopsies should be avoided due to haemorrhagic risk [2].

In diagnosing AVM in HHT syndrome, three scenarios can arise: First, when the diagnosis is confirmed and the patient shows clinical signs of HVM, their evaluation can help direct appropriate treatment. Second, when the diagnosis is suspected or unlikely based on the Curaçao criteria, identifying vascular malformations can help confirm the diagnosis. Third, when the diagnosis is confirmed but the patient shows no signs of HVM, systematic screening using color Doppler is conducted for early detection and proper management [3].

Currently, there is no definitive treatment for HHT. Management primarily focuses on symptom control, depending on the degree and type of involvement [2,3]. Most patients with symptomatic hepatic involvement can be treated medically, such as with antiangiogenic or antifibrinolytic agents. In some cases, liver transplantation may be necessary for refractory or advanced cases (high-output heart failure, biliary ischemia, or severe portal hypertension) [2,3]. Treatment with ursodeoxycholic acid may be considered depending on the case [3].

Individuals diagnosed with HHT or at risk due to family history should undergo regular follow-up evaluations [1,2]. An annual check-up is recommended to assess symptoms such as epistaxis, bleeding, shortness of breath, reduced exercise tolerance, headaches, or neurological issues [1]. Periodic monitoring of hematocrit and hemoglobin levels is necessary, with appropriate treatment for anemia if required. Reevaluation for pulmonary AVM should be conducted approximately every five years, using a contrast echocardiogram if no previous right-to-left shunt was detected or a chest CT if a shunt was previously identified.

## Conclusions

HHT is a rare vascular disorder with multisystem involvement. Hepatic vascular malformations, often asymptomatic, can lead to serious complications, including high-output cardiac failure, portal hypertension, and biliary involvement. Biliary complications result from ischemia due to arteriovenous or arterio-portal shunts, leading to strictures, cholangitis, cysts, necrosis, or bilomas. Imaging plays a key role in diagnosis and management. While no curative treatment exists, early detection and symptom-based care are essential to improve patient outcomes and quality of life.
